# Secreted phospholipase activity alters the post-secretory stability of extracellular vesicles

**DOI:** 10.1186/s13062-026-00937-7

**Published:** 2026-08-17

**Authors:** Gyeongmin Jeong, Rokgi Hong, Jeongwoo Yoo, Yu-bin Bae, Sungweon Ryoo, Byeong Geun Seok, Yang Jin, Seon-jin Lee, Jae-Min Lim, Younghoon Jang, Jun Go, Heedoo Lee

**Affiliations:** 1 Department of AI-Integrated Biological Sciences, Glocal Advanced Institute of Science & Technology - Artificial Intelligence (GAST-AI), Changwon National University, Changwon, 51140 Republic of Korea; 2 Department of Chemistry, College of Natural Sciences, Changwon National University, Changwon, 51140 Republic of Korea; 3 Biotechnology R&D Center, MycoDx Co., Ltd, Changwon, 51139 Republic of Korea; 4 Global Institute for Advanced Nanoscience & Technology (GIANT), Changwon National University, Changwon, 51140 Republic of Korea; 5 Division of Pulmonary and Critical Care Medicine, Department of Medicine, Boston University School of Medicine, Boston, MA 02118 USA; 6 University of Science and Technology, Daejeon, South Korea; 7 Environmental Disease Research Center, Korea Research Institute of Bioscience & Biotechnology, Daejeon, South Korea

**Keywords:** Extracellular vesicles, Secreted phospholipase activity, Secreted phospholipase A2, Post-secretory EV stability, Extracellular fluid

## Abstract

**Background:**

Extracellular vesicle (EV)-mediated signaling and EV-based delivery require that vesicles remain stable after release. In the post-secretory phase, released EVs encounter extracellular fluids before reaching recipient cells, yet the enzymatic mechanisms that modify EV stability in these environments remain poorly defined.

**Results:**

Using bronchoalveolar lavage fluid (BALF) as a physiologically relevant extracellular fluid model, we examined whether released EVs are enzymatically altered during the post-secretory extracellular phase. Exposure of EV preparations to BALF redistributed particle-size profiles toward smaller size fractions, increasing the 0–200 nm fraction from 26.1% to 46.1% and reducing the 200–400 nm fraction from 65.3% to 44.5%. This shift was reproduced by recombinant group V secreted phospholipase A2 (sPLA2) and attenuated by pharmacological sPLA2 inhibition, linking BALF-induced EV remodeling to sPLA2-sensitive phospholipid hydrolysis. Consistently, sPLA2 exposure generated an arachidonic acid (AA)-associated lipid signal, supporting EV membrane phospholipid hydrolysis. In vivo, adeno-associated virus-mediated expression of catalytically active sPLA2g5 shifted endogenous BALF EV profiles and reduced exogenous EV accumulation in BALF macrophages, whereas the catalytically inactive H67A mutant did not reproduce this effect. In lipopolysaccharide-induced pulmonary inflammation, Pla2g5 expression was increased and exogenous EV accumulation in BALF macrophages was reduced, and this reduction was attenuated by sPLA2 inhibition.

**Conclusions:**

These findings identify sPLA2-sensitive phospholipid hydrolysis as an extracellular mechanism that regulates post-secretory EV stability and indicate that phospholipase-active extracellular fluid environments can influence EV persistence and recipient-cell accumulation.

**Supplementary Information:**

The online version contains supplementary material available at https://doi.org/10.1186/s13062-026-00937-7.

## Introduction

Extracellular vesicles (EVs) are key mediators of intercellular communication that transfer bioactive cargos, including RNAs, proteins, and lipids, between cells [1, 2]. In addition to this role in endogenous communication, EVs have also emerged as promising therapeutic delivery platforms for diverse bioactive cargos [3, 4]. Because both EV-mediated signaling and EV-based cargo delivery depend on preservation of membrane integrity, structural stability is a fundamental prerequisite for EV function [5]. However, the mechanisms that regulate the stability of released EVs after secretion remain poorly defined.

This unresolved question is functionally important because productive EV signaling and cargo delivery depend on sufficient structural stability to preserve cargo, support membrane fusion and uptake, and enable effective transfer to recipient cells [5]. A quantitative analysis showed that EV uptake is intrinsically low-yield and that only a fraction of internalized EVs achieves productive content delivery [6]. For therapeutic applications, EVs also undergo rapid in vivo clearance [7]. These constraints highlight the extracellular interval between EV release and recipient-cell contact as a potentially important determinant of EV persistence and function. During this interval, released EVs are exposed to tissue fluid environments that contain lipids, soluble proteins, and enzymatic activities capable of modifying vesicle membranes [8–10]. This issue is particularly relevant in the respiratory system, where vesicles are exposed to bronchoalveolar lining fluid, a biochemically active extracellular milieu containing abundant phospholipids and lipid-remodeling enzymatic activities [11, 12]. In inflammatory lung disease, this fluid environment is further altered and phospholipase activity, including secreted phospholipase A2 (sPLA2) activity, is increased [12, 13].

Among these enzymatic activities, sPLA2 is a particularly plausible candidate regulator of post-secretory EV stability because it hydrolyzes membrane phospholipids. Beyond the respiratory system, sPLA2 is increased in several disease-associated extracellular fluids, including synovial fluid in arthritis [14] and cerebrospinal fluid in Alzheimer’s disease [15], and is also elevated in bronchoalveolar lavage fluid in inflammatory lung disease [12, 13, 16, 17]. Recent work in a non-pulmonary context further showed that sPLA2 can hydrolyze EV phospholipids and alter vesicle size and biological activity [10]. The relevance of this axis to the respiratory system is further supported by the identification of an sPLA2-IIA-associated EV pool in the BALF of patients with acute respiratory distress syndrome (ARDS) [18]. Collectively, these prior findings support the biological plausibility of sPLA2 as a candidate regulator of released EV stability in extracellular fluid environments, including the pulmonary extracellular space.

Based on these observations, we investigated post-secretory EV stability at the particle level, focusing on whether released EVs maintain their size profiles within extracellular fluid environments. We hypothesized that secreted phospholipase activity can alter this particle-level stability after EV release. We first used BALF as a physiologically relevant extracellular fluid model to determine whether extracellular fluid exposure changes the size distribution of released EVs. We next tested the role of sPLA2 activity by assessing the effects of recombinant group V sPLA2 and pharmacological sPLA2 inhibition on EV size redistribution, and measured arachidonic acid (AA) generation as a lipid-derived readout of phospholipid hydrolysis. We then evaluated the in vivo relevance of this mechanism by testing whether catalytically active sPLA2g5 alters endogenous BALF EV profiles and exogenous EV accumulation in BALF macrophages. Finally, we examined whether LPS-induced pulmonary inflammation engages this sPLA2–EV stability axis. Through this approach, we aimed to define extracellular sPLA2-sensitive phospholipid hydrolysis as a post-secretory mechanism that regulates released EV particle stability before recipient-cell accumulation.

## Methods

### Animals and in vivo treatments

Male C57BL/6N mice, 6–8 weeks of age, were obtained from Samtako Bio Korea (Osan, Republic of Korea) and maintained with free access to food and water. A single-sex male cohort was used to keep sex consistent across experimental groups in this initial mechanistic evaluation of the sPLA2-sensitive EV remodeling axis. Mice were housed at 20–24 °C under a 12 h light/dark cycle. All animal procedures were approved by the Institutional Animal Care and Use Committee of Changwon National University and were performed in accordance with the approved guidelines (approval number 2025-05).

For baseline characterization of the BALF EV enriched fraction, EV preparations were obtained from BALF collected from four untreated mice and used for nanoparticle tracking analysis and scanning electron microscopy. For BALF sPLA2 activity measurements and basal sPLA2 isoform expression analysis, BALF and matched lung tissues were obtained from four untreated mice. For the sPLA2 activity assay, BALF samples were pooled two-by-two, generating two pooled BALF samples. For each ex vivo EV incubation assay, EV preparations obtained from six untreated mice were divided into paired treatment conditions before incubation.

For AAV-mediated pulmonary sPLA2g5 overexpression, mice were acclimated for 1 week and used at 8 weeks of age. Mice were assigned to three groups, AAV-vector (*n* = 8), AAV-sPLA2g5-WT (*n* = 8), and AAV-sPLA2g5-H67A (*n* = 7). Each mouse received 1.0 × 10¹³ genome copies of the corresponding AAV9 vector in 80 µL by intratracheal administration. BALF and lung tissues were collected 2 weeks after AAV administration. Endogenous BALF EV profiles were analyzed using the full AAV cohort described above. For exogenous EV tracking, a subset of mice from each group (*n* = 4 per group) received PKH-labeled EVs 18 h before BALF collection, and macrophage-associated PKH-EV accumulation was quantified in this subset.

For the LPS-induced pulmonary inflammation experiment, mice were assigned to four groups, PBS (*n* = 6), AZD2716 (*n* = 6), LPS (*n* = 6), and LPS with AZD2716 (*n* = 6). PBS or LPS (L4268, Sigma-Aldrich, St. Louis, MO, USA) was administered intratracheally in 50 µL, with LPS used at 2 mg/kg, corresponding to 50 µg per mouse. Vehicle or AZD2716 (#35067, Cayman Chemical, Ann Arbor, MI, USA) was administered intratracheally in 50 µL, with AZD2716 used at 0.1 mg/mL, corresponding to 5 µg per mouse. Six hours after PBS or LPS exposure, PKH-labeled EVs were administered, and BALF and lung tissues were collected 6 h after PKH-labeled EV administration.

Intratracheal procedures were performed under isoflurane anesthesia (Hana Pharm Co., Ltd., Hwaseong, Korea). Mice were placed in an induction chamber and anesthetized with 3–5% isoflurane in oxygen using an R550 Multi-output Animal Anesthesia Machine (RWD Life Science Co., Ltd., Shenzhen, China). After adequate anesthesia was confirmed, the indicated agents were delivered intratracheally using a sterile pipette tip without surgical incision. Antibiotics were not administered. At each experimental endpoint, mice were euthanized by continuous exposure to isoflurane at ≥ 2% in oxygen until respiratory arrest was confirmed, and exposure was maintained for an additional period to ensure euthanasia before sample collection. BALF and lung tissues were then collected for downstream analyses.

### AAV vector construction, production, and in vitro validation

The pAAV-pEFS-IRES-EGFP vector was generated by cloning the pEFS promoter into the pAAV-IRES-EGFP backbone (VPK-418, Cell Biolabs, San Diego, CA, USA) using MluI and BsaBI restriction sites. To generate the catalytically inactive mutant, histidine 67, a catalytic residue of sPLA2g5, was substituted with alanine to produce the H67A mutant. The WT or H67A sPLA2g5 sequence fused with a 3xFLAG tag at the C-terminus was inserted into the AAV backbone using BamHI and EcoRI restriction sites, generating ITR-pEFS-sPLA2g5-3xFLAG-WT-IRES-EGFP-polyA-ITR and ITR-pEFS-sPLA2g5-3xFLAG-H67A-IRES-EGFP-polyA-ITR constructs. An empty pAAV-pEFS-IRES-EGFP vector was used as the vector control.

AAV Pro 293T cells (#632273, Takara Bio, Shiga, Japan) were maintained in DMEM supplemented with 10% fetal bovine serum and 1% antibiotic-antimycotic solution at 37 °C in a humidified 5% CO₂ incubator. For AAV9 production, cells were seeded in 150 mm dishes at 5.0 × 10⁶ cells per dish. Forty-two hours after seeding, cells were co-transfected with each AAV transfer plasmid together with pHelper (#112867, Addgene, Watertown, MA, USA) and pAAV2/9n (#112865, Addgene) using polyethyleneimine (Polysciences, Warrington, PA, USA). Specifically, 4.93 µg of AAV-sPLA2g5-H67A, 4.93 µg of AAV-sPLA2g5-WT, or 4.56 µg of AAV-vector plasmid was co-transfected with 12.63 µg of pHelper, 6 µg of pAAV2/9n, and 70 µg of polyethyleneimine. The medium was replaced 24 h after transfection, and cells were cultured for an additional 48 h. AAV particles were harvested according to an established protocol, and viral titers were determined by qPCR using ITR-specific primers listed in Supplementary Table 1.

For in vitro validation of AAV construct expression, AAV Pro 293T cells were seeded in 6-well plates at 2.4 × 10⁵ cells per well and transfected when they reached approximately 70% confluence. Cells were co-transfected with 3 µg of AAV-sPLA2g5-H67A, AAV-sPLA2g5-WT, or AAV-vector plasmid together with 0.9 µg of pHelper, 0.4 µg of pAAV2/9n, and 1.6 µg of polyethyleneimine. The medium was replaced 24 h after transfection, and cells were harvested after an additional 24 h of culture. Expression of FLAG-tagged sPLA2g5, GFP, and β-actin was assessed by Western blotting.

### BALF collection, EV preparation, and ex vivo EV incubation

BALF was collected by tracheal cannulation and lavage with DPBS (BS11000A, Bandio, Pocheon, Korea). BALF samples were centrifuged at 500 × g for 5 min at 4 °C to remove cells. The cell-free BALF supernatant was then centrifuged at 10,000 × g for 30 min at 4 °C to obtain a BALF EV-enriched fraction. The resulting BALF EV preparation was resuspended in DPBS and used for nanoparticle tracking analysis, scanning electron microscopy, PKH labeling, or ex vivo incubation assays.

For paired ex vivo comparisons, each mouse-derived EV-enriched preparation was divided into two treatment conditions before incubation. For BALF exposure assays, EV preparations were incubated in PBS or BALF at 37 °C for 18 h. For recombinant sPLA2 treatment, EV preparations in PBS were mixed with an equal volume of PBS or recombinant human group V sPLA2 (#10009563, Cayman Chemical; final concentration, 125 µg/mL) and incubated at 37 °C for 18 h. For inhibitor experiments, EV preparations were incubated in BALF in the presence or absence of AZD2716 at 0.83 mg/mL for 18 h at 37 °C. For combined recombinant sPLA2 and inhibitor experiments, isolated EVs were incubated with recombinant group V sPLA2 in the presence or absence of AZD2716 at 37 °C for 18 h. After incubation, EV preparations were recovered by centrifugation at 10,000 × g for 30 min at 4 °C, resuspended in DPBS, and subjected to nanoparticle tracking analysis, scanning electron microscopy as indicated. For arachidonic acid analysis, EV incubation samples were centrifuged at 10,000 × g for 30 min at 4 °C to remove EVs, and the supernatants were collected for UHPLC-MS analysis as described below.

### PKH-labeled EV preparation and BALF macrophage analysis

BALF EV preparations containing 6.4 × 10⁹ particles were labeled with 5 µL of PKH26 dye (MINI26, Sigma-Aldrich, St. Louis, MO, USA) in a final labeling volume of 500 µL for 30 min at room temperature. Following labeling, the samples were centrifuged at 10,000 × g for 30 min at 4 °C. The resulting pellet was washed three times with DPBS by repeated resuspension and centrifugation under the same conditions to remove unbound dye. After the final wash, the PKH-labeled EV preparation was resuspended in 100 µL of DPBS, and 50 µL of the suspension was administered intratracheally to each of two mice. A PKH-only control was prepared in parallel without EVs and subjected to the same dye incubation, centrifugation, washing, resuspension, and intratracheal administration procedures. BALF cells collected after PKH-labeled EV or PKH-only control administration were centrifuged at 1,200 rpm for 20 min and prepared on cytospin slides using a Cell Spin II cytocentrifuge (Hanil, Gimpo, Korea). Slides were counterstained with DAPI (F6057, Sigma-Aldrich).

BALF macrophages were identified based on cytospin morphology, using larger macrophage-like cells with abundant cytoplasm as the macrophage population and excluding smaller polymorphonuclear neutrophil-like cells. This morphology-based approach is consistent with standard BALF cytospin differential cell counting, in which macrophages/monocytes and neutrophils are distinguished by cytological morphology [19, 20]. BALF cytospin slides from PBS- or LPS-treated mice were stained with hematoxylin and eosin (H&E), and macrophage-like and neutrophil-like cells were counted per field using these morphological criteria (Supplementary Fig. 1). Because BALF under non-inflammatory conditions is normally dominated by alveolar macrophages [21, 22], macrophage-associated PKH-EV accumulation in the AAV experiment was quantified in morphologically identified BALF macrophages. Cell numbers per field and PKH-EV⁺ area within macrophages were quantified using ImageJ software (National Institutes of Health, Bethesda, MD, USA). In the LPS experiment, PKH-EV⁺ area was quantified within macrophage regions rather than across the total field to minimize the influence of increased non-macrophage inflammatory cells in BALF cytospin images.

### Nanoparticle tracking analysis

Particle concentration and size distribution were measured using an NS300 nanoparticle tracking analysis instrument (Malvern Panalytical Ltd., Malvern, UK). EV preparations were diluted in DPBS to generate 100-fold and 1,000-fold dilutions. Two diluted samples were prepared for each group, and each sample was analyzed five times using a syringe pump. Instrument settings were kept constant during acquisition. Camera level was set to 5, screen gain to 8, and detection threshold to 3. Particle size distributions were analyzed by dividing particles into 0–200 nm, 200–400 nm, and 400–1,000 nm fractions.

### Scanning electron microscopy

For scanning electron microscopy, 10 µL of EV preparation was applied onto a scanning electron microscopy stub. Samples were air-dried in an oven and fixed with 10 µL of 3.7% formaldehyde for 5 min at room temperature. After fixation, samples were washed with DPBS and dried again. EV morphology was observed using a field-emission scanning electron microscope (JSM-7900F, JEOL, Tokyo, Japan), at the indicated magnifications.

### sPLA2 activity assay

sPLA2 activity in BALF was measured using a commercial sPLA2 activity assay kit (#765001, Cayman Chemical) according to the manufacturer’s protocol. Each reaction contained 10 µL of BALF sample, 5 µL of sPLA2 assay buffer, 10 µL of DTNB, and 200 µL of diheptanoyl thio-PC substrate solution. Absorbance was measured at 414 nm using a microplate reader (Multiskan SkyHigh, Thermo Fisher Scientific, Waltham, MA, USA), and the enzymatic reaction signal was presented as optical density (O.D.).

### Western blotting

Cells were lysed in RIPA buffer supplemented with 100× protease inhibitor cocktail (Abbkine, Wuhan, China) and 100× phosphatase inhibitor cocktail (ChemScene, Monmouth Junction, NJ, USA). Lysates were incubated on ice for 20 min and centrifuged at 12,500 × g for 15 min at 4 °C. Protein-containing supernatants were collected, mixed with NuPAGE LDS sample buffer (Thermo Fisher Scientific) at a 3:1 ratio, and heated at 97 °C for 8 min. Proteins were separated by SDS-PAGE and transferred onto PVDF membranes (Thermo Fisher Scientific). Membranes were incubated with primary antibodies against FLAG (#14793, Cell Signaling Technology, Danvers, MA, USA), GFP (#2956, Cell Signaling Technology), and β-actin (#4970, Cell Signaling Technology) diluted 1:1000 in 3% BSA, followed by HRP-conjugated anti-rabbit IgG secondary antibody (#7074, Cell Signaling Technology) diluted 1:10,000 in 3% skim milk. Protein bands were visualized using ECL chemiluminescence reagent (Thermo Fisher Scientific) and an iBright 1500 imaging system (Thermo Fisher Scientific).

### Quantitative real-time PCR

Total RNA was extracted from lung tissues using easy-BLUE Total RNA Extraction Kit (Cat. No. 17221, iNtRON Biotechnology, Seongnam, Korea). cDNA was synthesized using High-Capacity cDNA Reverse Transcription Kit (#4368813, Thermo Fisher Scientific) according to the manufacturer’s instructions. Quantitative real-time PCR was performed using PowerUp™ SYBR™ Green Master Mix for qPCR (Cat. No. A25778, Thermo Fisher Scientific) on a QuantStudio 1 Real-Time PCR system (Thermo Fisher Scientific). Relative mRNA expression was calculated using the 2 − ΔΔCt method with *Actb* as the internal control. Expression of *Pla2g2a*, *Pla2g2d*, *Pla2g3*, *Pla2g5*, and *Pla2g10* was analyzed in lung tissues. For AAV transgene validation, qPCR was performed using a WT-specific primer set and a second primer set shared by the WT and H67A constructs. Primer sequences are listed in Supplementary Table 1.

### Arachidonic acid analysis by ultra-high-performance liquid chromatography–mass spectrometry

Arachidonic acid was analyzed as a lipid-derived readout of sPLA2-mediated phospholipid hydrolysis. For experimental samples, three independent EV preparations were divided into four treatment conditions and incubated in DPBS alone, DPBS containing recombinant group V sPLA2, DPBS containing AZD2716, or DPBS containing both recombinant group V sPLA2 and AZD2716 at 37 °C for 18 h. After incubation, samples were centrifuged at 10,000 × g for 30 min at 4 °C to remove EVs, and the supernatants were collected for analysis.

For sample preparation, 200 µL of each supernatant was mixed with 800 µL of 100% methanol to obtain a final volume of 1 mL containing 80% methanol. Arachidonic acid standard solutions were prepared at 0.05, 0.1, 0.5, and 1.0 ppm. Ultra-high-performance liquid chromatography–mass spectrometry was performed using an UltiMate 3000 UHPLC system coupled to a Q Exactive Plus Orbitrap mass spectrometer equipped with a heated electrospray ionization source (Thermo Fisher Scientific, Waltham, MA, USA) at the Gyeongnam Bio and Anti-aging Core Facility Center for CWNU.

Chromatographic separation was performed using a reversed-phase XBridge BEH C18 column (3.5 μm, 2.1 × 50 mm; Waters, Milford, MA, USA). A 10 µL aliquot of each sample was injected using an autosampler. The mobile phases consisted of water as solvent A and 100% methanol as solvent B at a flow rate of 200 µL/min. The gradient elution program was as follows: 10% B from 0 to 2 min, 30% B from 2 to 8 min, 50% B from 8 to 12 min, 70% B from 12 to 15 min, 99% B from 15 to 20 min, followed by re-equilibration at 0% B from 20 to 30 min.

Mass spectrometric data were acquired in negative ionization mode under the following conditions: capillary voltage, 3.0 kV; capillary temperature, 250 °C; and S-lens RF level, 50. Full-scan Fourier transform mass spectra were acquired in profile mode at a resolution of 70,000 over an m/z range of 100–700, with two microscans and a maximum injection time of 150 ms. Tandem mass spectrometry analysis for structural confirmation was performed using higher-energy collisional dissociation with a normalized collision energy of 20%. Arachidonic acid-associated ions were evaluated near m/z 303.232.

### Statistical analysis

Data are presented as mean ± SD. Statistical analyses were performed using GraphPad Prism 8.0.2. Paired two-tailed Student’s t-tests were used for split-sample ex vivo EV incubation assays. Repeated-measures one-way ANOVA followed by multiple-comparison testing was used for matched size-fraction comparisons. Unpaired two-tailed Student’s t-tests were used for comparisons between two independent groups, and one-way ANOVA followed by Tukey’s multiple comparison test was used for comparisons among three or more independent groups. *p* < 0.05 was considered statistically significant. Exact P values are provided in Supplementary Table 2.

## Results

### Extracellular fluid exposure redistributes released extracellular vesicle size profiles

**Fig. 1:**
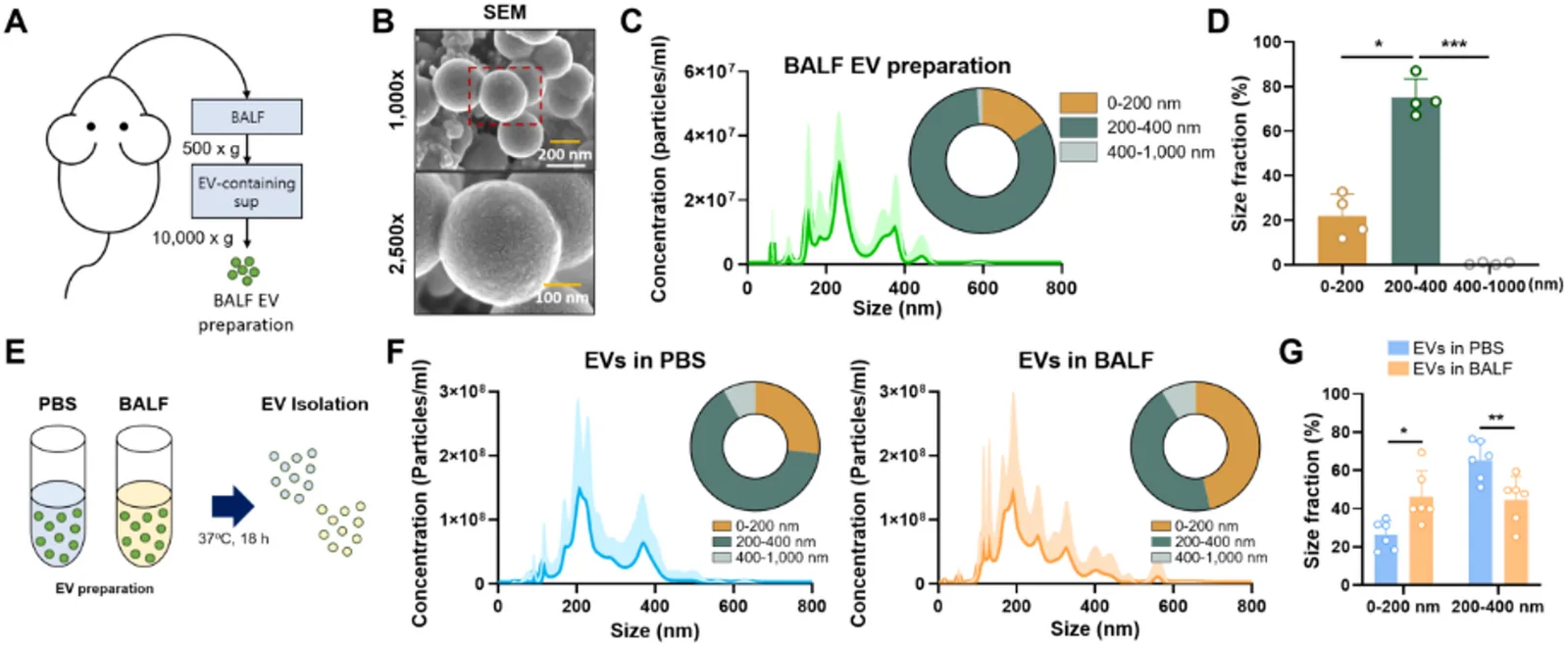
Bronchoalveolar lavage fluid alters the size distribution of released extracellular vesicles. (**A**) Schematic overview of preparation of an extracellular vesicle (EV)-enriched fraction from bronchoalveolar lavage fluid (BALF) by differential centrifugation. (**B**) Representative scanning electron microscopy images of the BALF EV preparation. Scale bars, 200 nm and 100 nm. (**C**) Representative nanoparticle tracking analysis profile of the BALF EV preparation and the corresponding size-fraction distribution. (**D**) Quantification of size fractions in the BALF EV preparation, *n* = 4 mice. (**E**) Schematic of ex vivo EV exposure to phosphate-buffered saline (PBS) or BALF, *n* = 6 mouse-derived EV preparations split into paired PBS and BALF exposure conditions. (**F**) Representative nanoparticle tracking analysis profiles and size-fraction distributions of EV preparations after PBS or BALF exposure. (**G**) Quantification of EV size fractions after PBS or BALF exposure. Data are presented as mean ± SD. Statistical significance was determined by repeated-measures one-way ANOVA with multiple-comparison testing in (**D**) and by paired two-tailed Student’s t-tests in (**G**). Statistical significance is indicated as *p < 0.05, **p < 0.01, and ***p < 0.005

To evaluate whether extracellular fluid exposure can alter released extracellular vesicles (EVs), we first prepared BALF EV-enriched fraction by differential centrifugation and characterized their morphology and size distribution (Fig. 1A). Scanning electron microscopy showed rounded vesicle-like structures, and nanoparticle tracking analysis confirmed a heterogeneous particle population (Fig. 1B and C). Size-fraction analysis further showed that the measured particles were predominantly detected in the 200–400 nm range, with smaller proportions in the 0–200 nm and 400–1,000 nm ranges (Fig. 1D).

We next examined whether exposure to BALF after EV isolation was sufficient to alter the size distribution of released EVs. To this end, EV preparations were incubated in PBS or BALF at 37 °C for 18 h and subsequently reanalyzed by nanoparticle tracking analysis (Fig. 1E and F). Compared with PBS-incubated EV preparations, BALF-exposed EV preparations exhibited size redistribution toward smaller particles, with an increased proportion of particles in the 0–200 nm range and a reduced proportion in the 200–400 nm range (Fig. 1G). These findings indicate that exposure to BALF is sufficient to alter released EV particle size profiles, supporting the concept that extracellular fluid conditions can alter EV size profiles during the post-secretory phase.

### Secreted phospholipase A2 activity contributes to extracellular fluid-induced EV size redistribution

**Fig. 2:**
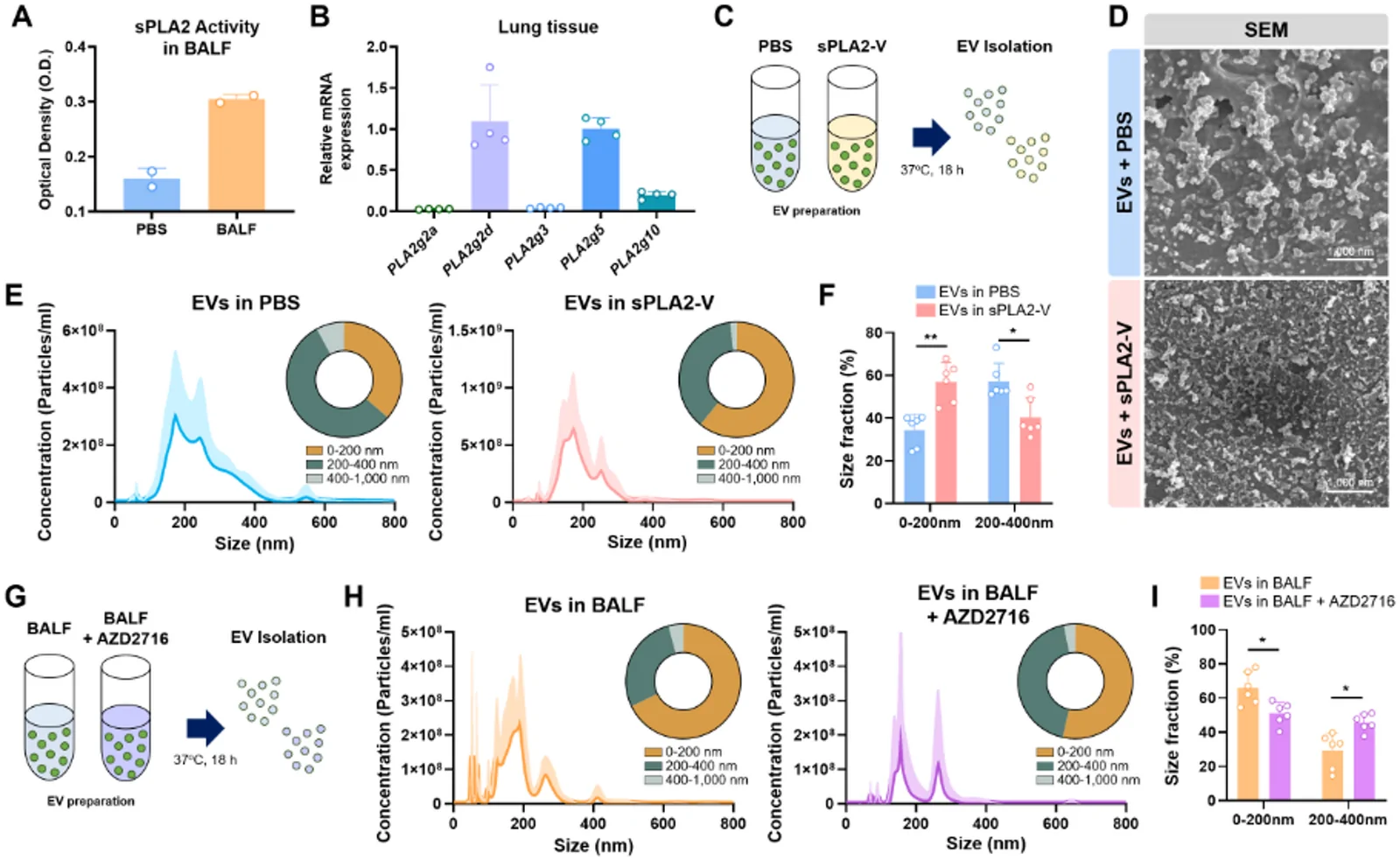
Secreted phospholipase A2 activity contributes to extracellular vesicle size redistribution. (**A**) Secreted phospholipase A2 (sPLA2) enzymatic reaction signal in phosphate-buffered saline (PBS) and bronchoalveolar lavage fluid (BALF), *n* = 2 pooled BALF samples, each generated by pooling BALF from two mice. (**B**) Relative mRNA expression of sPLA2 isoforms in matched lung tissue from the same mice, *n* = 4 mice. (**C**) Schematic of exposure of extracellular vesicle (EV) preparations to PBS or recombinant group V sPLA2 (sPLA2-V), *n* = 6 mouse-derived EV preparations split into paired PBS and sPLA2-V exposure conditions. (**D**) Representative scanning electron microscopy images of EV preparations after PBS or sPLA2-V exposure. Scale bars, 1,000 nm. (**E**) Representative nanoparticle tracking analysis profiles and size-fraction distributions of EV preparations after PBS or sPLA2-V exposure. (**F**) Quantification of size fractions after PBS or sPLA2-V exposure. (**G**) Schematic of exposure of EV preparations to BALF with or without AZD2716, *n* = 6 mouse-derived EV preparations split into paired BALF and BALF + AZD2716 exposure conditions. (**H**) Representative nanoparticle tracking analysis profiles and size-fraction distributions of EV preparations after BALF exposure with or without AZD2716. (**I**) Quantification of size fractions after BALF exposure with or without AZD2716. Data are presented as mean ± SD. Statistical significance was determined by paired two-tailed Student’s t-tests in (**F**) and (**I**). Statistical significance is indicated as *p < 0.05, **p < 0.01

Because BALF exposure shifted released EVs toward smaller-size populations (Fig. 1), we next examined whether this extracellular fluid-induced redistribution involved sPLA2 activity. As an initial biochemical readout of this possibility, we measured sPLA2 activity and detected an enzymatic signal in BALF (Fig. 2A). We then quantified the expression of sPLA2 isoforms in lung tissue, including *Pla2g2a*, *Pla2g2d*, *Pla2g3*, *Pla2g5*, and *Pla2g10*. Among the isoforms examined in this qPCR panel, *Pla2g2d* and *Pla2g5* showed comparatively prominent transcript signals, whereas *Pla2g2a*, *Pla2g3*, and *Pla2g10* showed lower signals (Fig. 2B). These results, together with prior evidence implicating group V sPLA2 in airway epithelial biology and acute lung injury, led us to focus on group V sPLA2 in subsequent functional experiments [23, 24].

To determine whether group V sPLA2 is sufficient to recapitulate the extracellular fluid-associated shift in EV size distribution, isolated EVs were incubated with PBS or recombinant sPLA2-V at 37 °C for 18 h, followed by EV recovery and analysis (Fig. 2C). Scanning electron microscopy was used to visualize EV preparations after PBS or sPLA2-V exposure (Fig. 2D). Nanoparticle tracking analysis showed that sPLA2-V treatment shifted EVs toward smaller-size particles, with an increased 0–200 nm fraction and a reduced 200–400 nm fraction compared with PBS-treated EVs (Fig. 2E and F).

We next tested whether inhibition of sPLA2 activity attenuates BALF-induced EV size redistribution. EVs were incubated in BALF in the presence or absence of the pharmacological sPLA2 inhibitor AZD2716 at 37 °C for 18 h, followed by EV recovery and nanoparticle tracking analysis (Fig. 2G and H). AZD2716 attenuated the BALF-induced shift toward smaller particles, as shown by a reduced 0–200 nm fraction and an increased 200–400 nm fraction compared with EVs incubated in BALF alone (Fig. 2I). These findings indicate that sPLA2 activity contributes to extracellular fluid-induced EV size redistribution and that group V sPLA2 is sufficient to reproduce this shift in EV size distribution.

### sPLA2-mediated EV size redistribution is accompanied by arachidonic acid generation

**Fig. 3:**
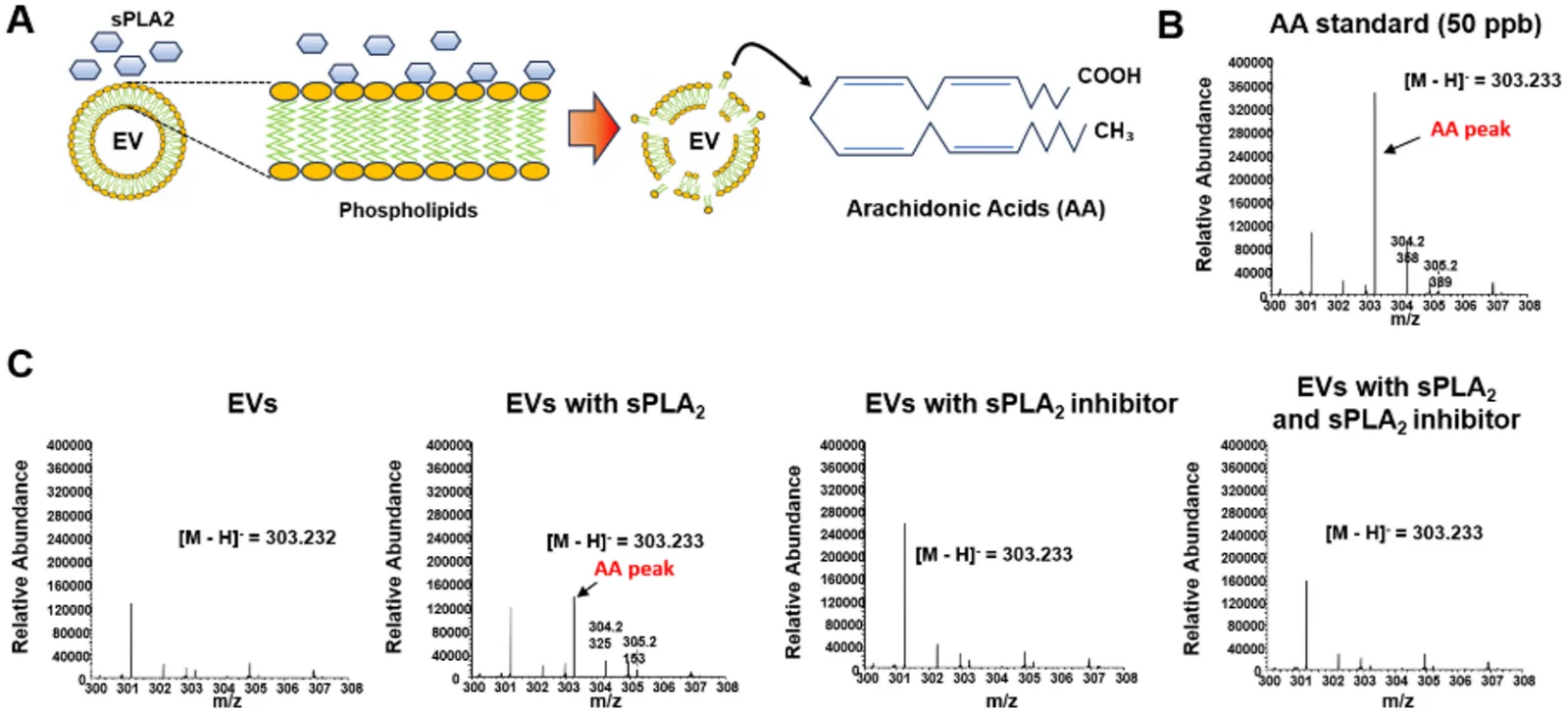
sPLA2-mediated extracellular vesicle size redistribution is accompanied by arachidonic acid generation. (**A**) Schematic illustration of secreted phospholipase A2 (sPLA2)-mediated phospholipid hydrolysis and arachidonic acid (AA) generation from extracellular vesicle (EV) membranes. (**B**) Representative UHPLC-MS spectrum of the AA standard. (**C**) Representative UHPLC-MS spectra of supernatants from EV preparations incubated alone, with sPLA2, with the sPLA2 inhibitor, or with both sPLA2 and the sPLA2 inhibitor. An AA-associated signal was detected in supernatants from sPLA2-treated EV preparations, and representative spectra are shown from one of three independent EV preparations per condition

After establishing that group V sPLA2 was sufficient to shift EV size distribution (Fig. 2), we next examined whether this response was accompanied by biochemical evidence of EV membrane phospholipid hydrolysis. Because sPLA2 enzymes hydrolyze the sn-2 ester bond of glycerophospholipids and can generate free fatty acids, including arachidonic acid (AA), we used AA as a lipid-derived readout of phospholipid hydrolysis during EV size redistribution (Fig. 3A and B) [25, 26]. Mass spectrometric analysis of an AA standard identified the expected deprotonated AA ion near m/z 303.2322 (Fig. 3B). In EV incubation supernatants, samples from sPLA2-treated EV preparations showed a more evident AA-associated signal near m/z 303.2326, compared with EVs alone or EVs treated with the sPLA2 inhibitor alone (Fig. 3C). This AA-associated signal was reduced when EVs were cotreated with sPLA2 and the sPLA2 inhibitor (Fig. 3C). These findings indicate that sPLA2-mediated particle-size redistribution occurred together with AA generation, providing a lipid-derived biochemical readout of sPLA2-sensitive phospholipid hydrolysis during post-secretory particle remodeling.

### Catalytically active sPLA2g5 alters endogenous EV profiles and limits macrophage-associated EV accumulation in vivo

**Fig. 4:**
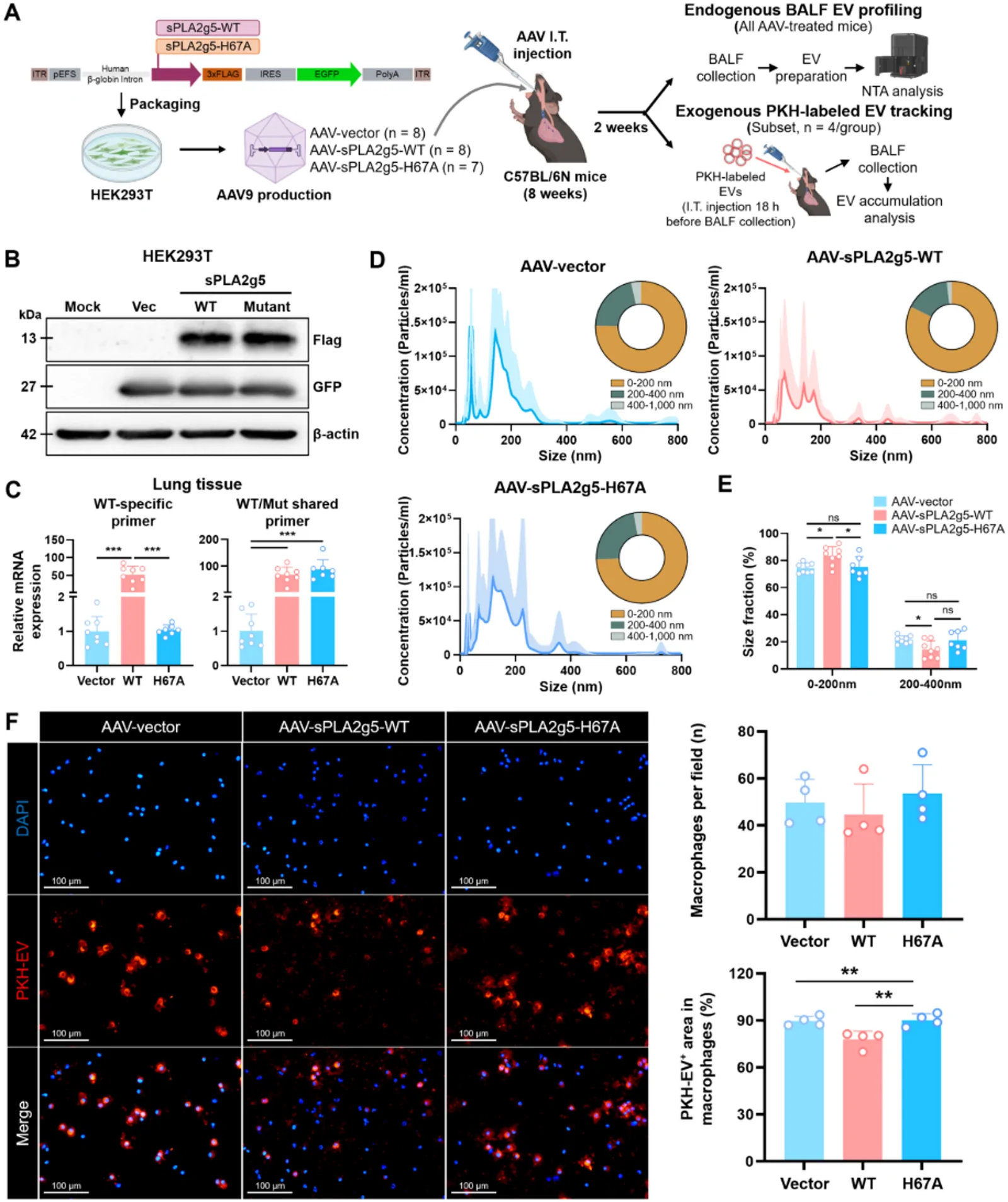
Catalytically active sPLA2g5 alters BALF EV profiles and macrophage-associated EV accumulation. (**A**) Schematic overview of AAV9-mediated pulmonary expression of wild-type sPLA2g5 or catalytically inactive sPLA2g5-H67A and subsequent analyses of endogenous extracellular vesicle (EV) preparations from bronchoalveolar lavage fluid (BALF) and exogenous PKH-labeled EVs. (**B**) Western blot validation of FLAG-tagged sPLA2g5 and GFP expression in HEK293T cells. β-actin was used as a loading control. (**C**) qPCR validation of WT-specific and WT/Mut shared sPLA2g5 transgene expression in lung tissue, *n* = 8, 8, and 7 mice for AAV-vector, AAV-sPLA2g5-WT, and AAV-sPLA2g5-H67A, respectively. (**D**) Representative nanoparticle tracking analysis profiles and size-fraction distributions of endogenous BALF EV preparations from AAV-vector, AAV-sPLA2g5-WT, and AAV-sPLA2g5-H67A mice. (**E**) Quantification of size fractions in endogenous BALF EV preparations. (**F**) Representative BALF cytospin images after exogenous PKH-labeled EV administration and quantification of macrophage numbers and PKH-EV⁺ area in macrophages, *n* = 4 mice per group from the same AAV-treated cohort. Data are presented as mean ± SD. Statistical significance was determined by one-way ANOVA with Tukey’s multiple comparison test in (**C**), (**E**), and (**F**). Statistical significance is indicated as *p < 0.05, **p < 0.01, and ***p < 0.005; ns, not significant. Uncropped Western blot images are provided in Supplementary Fig. 3

To determine whether sPLA2g5 can alter released EVs in vivo, we generated AAV9 vectors expressing wild-type sPLA2g5 or a catalytically inactive H67A mutant and administered them intratracheally to C57BL/6N mice (Fig. 4A). In HEK293T cells, Western blot analysis confirmed the expected expression pattern of the AAV constructs. FLAG-tagged sPLA2g5 was detected only in cells receiving the WT or H67A mutant construct, whereas GFP was detected in cells receiving the empty vector, WT, or H67A mutant construct, with β-actin used as a loading control (Fig. 4B). In lung tissue, the WT-specific primer detected increased sPLA2g5 expression only in the AAV-sPLA2g5-WT group, whereas the WT/Mut shared primer confirmed increased transgene expression in both the AAV-sPLA2g5-WT and AAV-sPLA2g5-H67A groups (Fig. 4C). Together, these results verified construct expression in vitro and confirmed in vivo expression of the WT and H67A sPLA2g5 transgenes.

To test whether catalytically active sPLA2g5 altered endogenous EV profiles in vivo, we analyzed endogenous BALF EV preparations obtained 2 weeks after AAV administration. Nanoparticle tracking analysis showed that AAV-sPLA2g5-WT shifted the particle-size profiles of endogenous BALF EV preparations toward smaller particles, as reflected by an increased 0–200 nm fraction and a reduced 200–400 nm fraction compared with the AAV-vector control (Fig. 4D and E). The H67A mutant showed a less pronounced increase in the 0–200 nm fraction than AAV-sPLA2g5-WT, consistent with a contribution of sPLA2g5 catalytic activity to endogenous EV size redistribution in vivo (Fig. 4E).

We next asked whether this catalytic effect was associated with reduced accumulation of exogenous EVs in recipient cells. PKH-labeled EVs were delivered to AAV-treated mice, and BALF macrophages were analyzed 18 h later as a recipient phagocytic cell population exposed to intratracheally delivered EVs (Fig. 4A and F) [27]. To assess residual dye carryover from the labeling procedure, a PKH-only control was processed and administered using the same procedure as PKH-labeled EVs. This control showed little detectable PKH fluorescence in BALF cytospin preparations, indicating that residual dye carryover was minimal under these conditions (Supplementary Fig. 2). The number of macrophages per field was not significantly altered by AAV-mediated overexpression of WT or H67A sPLA2g5 compared with the vector control (Fig. 4F). In contrast, the PKH-EV⁺ area in macrophages was reduced in the AAV-sPLA2g5-WT group, whereas this reduction was not observed in the AAV-sPLA2g5-H67A group (Fig. 4F). Together, these findings indicate that catalytically active sPLA2g5 alters endogenous BALF EV size profiles and limits exogenous EV accumulation in BALF macrophages in vivo, supporting an enzymatic contribution to post-secretory EV stability.

### LPS-induced inflammation reduces exogenous EV accumulation in an sPLA2-sensitive manner

**Fig. 5:**
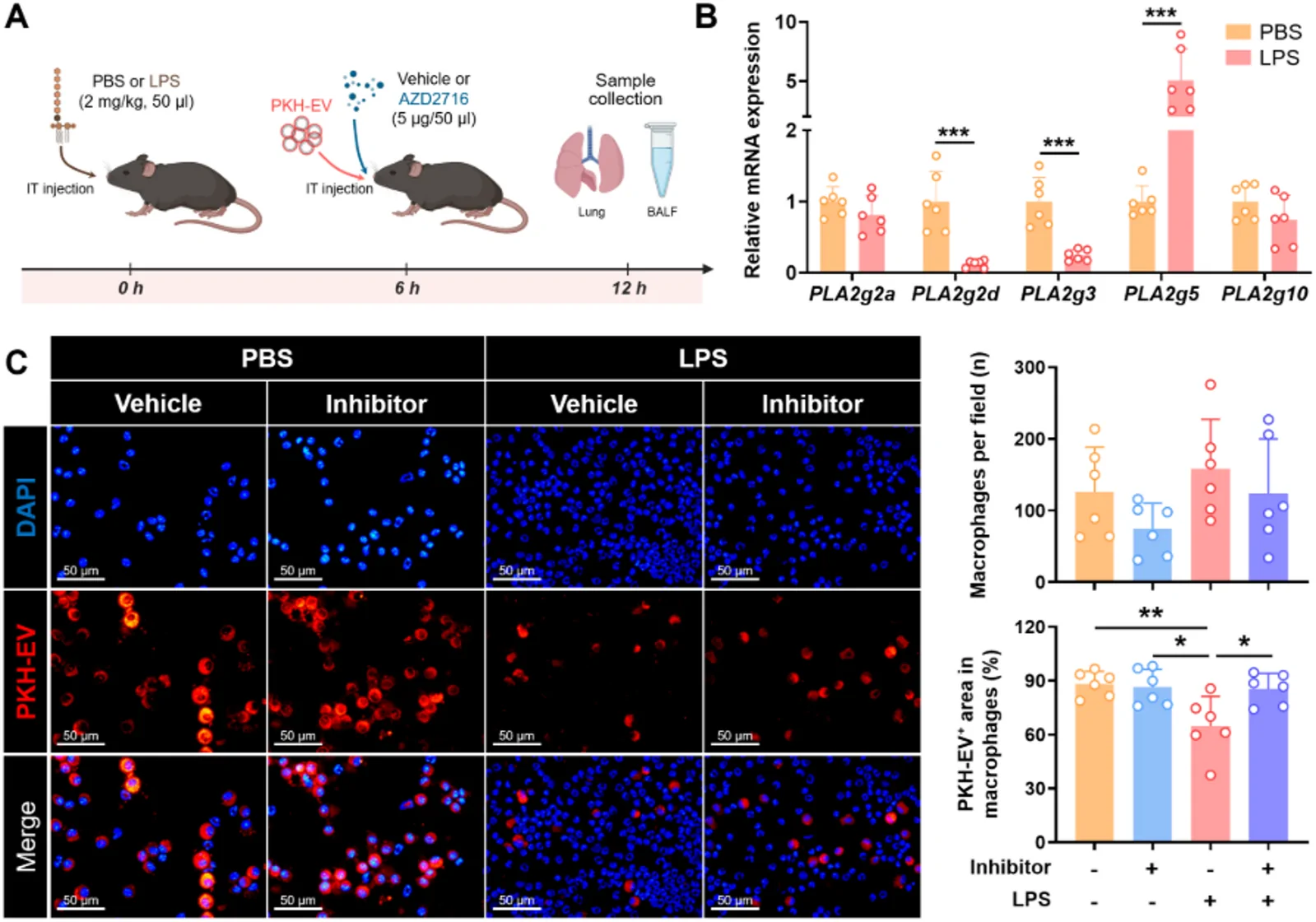
sPLA2 inhibition preserves exogenous EV accumulation in BALF macrophages during LPS-induced pulmonary inflammation. (**A**) Experimental scheme for assessing the effect of sPLA2 inhibition on exogenous EV accumulation during LPS-induced pulmonary inflammation. (**B**) Relative mRNA expression of sPLA2 isoforms in lung tissue after PBS or LPS administration, *n* = 6 mice per group. (**C**) Representative BALF cytospin images after PKH-labeled EV administration and quantification of macrophage numbers and PKH-EV⁺ area in macrophages, *n* = 6 mice per group. Data are presented as mean ± SD. Statistical significance was determined by unpaired two-tailed Student’s t-test in (**B**) and by one-way ANOVA with Tukey’s multiple comparison test in (**C**). Statistical significance is indicated as *p < 0.05, **p < 0.01, and ***p < 0.005

Having shown that extracellular fluid exposure and sPLA2 activity alter released EV size profiles, and that catalytically active sPLA2g5 limits exogenous EV accumulation in BALF macrophages in vivo (Figs. 1, 2, 3 and 4), we next examined whether this sPLA2-sensitive EV stability axis is engaged during LPS-induced inflammation. Mice were exposed to PBS or LPS, treated with vehicle or the pharmacological sPLA2 inhibitor AZD2716, and then administered PKH-labeled EVs before BALF and lung analysis (Fig. 5A). In LPS-exposed lungs, qPCR analysis showed increased Pla2g5 expression, whereas Pla2g2d and Pla2g3 expression were reduced and Pla2g2a and Pla2g10 were not significantly changed (Fig. 5B). This expression pattern identified Pla2g5 upregulation as a prominent sPLA2-related feature of this inflammatory condition.

We then analyzed exogenous EV accumulation in BALF macrophages after PKH-labeled EV administration (Fig. 5C). Because LPS exposure produced a prominent influx of neutrophil-like non-macrophage inflammatory cells in BALF cytospins, as shown by cytospin morphology in Supplementary Fig. 1, exogenous EV accumulation was quantified specifically within macrophages rather than across the total field (Fig. 5C). Macrophage numbers per field were comparable across the four treatment conditions (Fig. 5C). In contrast, the PKH-EV⁺ area in macrophages was reduced in LPS-exposed mice compared with PBS-treated mice, whereas AZD2716 treatment attenuated this reduction in LPS-exposed mice (Fig. 5C). Together, these findings indicate that LPS-induced inflammation engages an sPLA2-sensitive process that limits exogenous EV accumulation in recipient macrophages, extending post-secretory EV stability regulation to an inflammatory in vivo context.

## Discussion

This study identifies sPLA2 activity as an extracellular regulator of post-secretory EV stability. Our findings show that released EVs undergo size redistribution after BALF exposure and that sPLA2 treatment is accompanied by an arachidonic acid-associated lipid signal. In vivo, catalytically active sPLA2g5 reduced exogenous EV accumulation in recipient macrophages, and LPS-induced inflammation produced a similar reduction that was attenuated by sPLA2 inhibition. Together, these findings place the extracellular fluid encountered before recipient-cell contact as an active determinant of EV persistence and cellular accumulation. This post-secretory perspective expands the interpretation of EV-mediated communication and EV-based delivery beyond donor-cell release, vesicle cargo, biodistribution, and recipient-cell uptake [5–7].

These findings can be considered at three analytical levels of post-secretory EV stability. At the particle level, stability was assessed by maintenance or redistribution of NTA-based particle-size profiles after exposure to BALF, recombinant sPLA2, or sPLA2-active in vivo conditions. At the biochemical level, the AA-associated lipid signal provided evidence of sPLA2-mediated phospholipid hydrolysis accompanying EV remodeling. At the recipient-cell level, macrophage-associated PKH-EV fluorescence provided a cellular accumulation readout, indicating how extracellular EV remodeling relates to the accumulation of administered EVs in target phagocytic cells. Together, these readouts characterize particle-level remodeling, the accompanying biochemical signal, and macrophage-associated EV accumulation under sPLA2-active conditions.

Prior studies provide an important context for interpreting this sPLA2-sensitive EV stability mechanism. In lymphoma, sPLA2-mediated hydrolysis of EV phospholipids generated fatty acids, lysophospholipids, and downstream lipid metabolites, producing smaller EVs with altered macrophage uptake and protumorigenic activity [10]. In human ARDS, sPLA2-IIA protein and mRNA were detected in BALF EV fractions, indicating that inflammatory airway fluids can contain EV-associated sPLA2 signals [18]. Together, these studies establish EV membranes as sPLA2-responsive lipid substrates and show that sPLA2-associated EV pools can occur in inflammatory extracellular fluids. The present study links these concepts to post-secretory EV stability by showing that sPLA2-sensitive extracellular activity alters released EV size profiles in vivo. The AA-associated signal observed after sPLA2 exposure further provides a lipid-derived biochemical readout of sPLA2-sensitive phospholipid hydrolysis accompanying post-secretory particle remodeling, consistent with the established sPLA2 reaction at the sn-2 position of glycerophospholipids [25, 26].

Among the sPLA2 isoforms examined, sPLA2g5 emerged as the isoform most closely aligned with the inflammatory condition in which exogenous EV accumulation was reduced. Although *Pla2g5* and *Pla2g2d* showed comparatively prominent transcript signals in the basal lung qPCR panel, the LPS response provided a more informative inflammatory context. LPS exposure did not induce a uniform sPLA2 isoform response. Instead, *Pla2g5* was increased, whereas *Pla2g2d* and *Pla2g3* were reduced and *Pla2g2a* and *Pla2g10* remained unchanged. This interpretation is consistent with prior reports that group V sPLA2 is present in human airway epithelium and contributes to LPS-induced acute lung injury and neutrophilic inflammation in mice [23, 24]. The WT and H67A comparison further linked this response to enzymatic activity. The H67A mutant disrupts the catalytic histidine residue while preserving transgene expression, allowing catalytic activity to be separated from sPLA2g5 expression. Only catalytically active WT sPLA2g5 reduced exogenous EV accumulation in BALF macrophages. These data support a catalytic role for sPLA2g5 in regulating post-secretory EV stability and recipient-cell accumulation in vivo.

The reduction in macrophage-associated EV fluorescence also has implications for interpreting EV delivery after exposure to enzymatically active extracellular fluids. EV delivery studies commonly evaluate post-administration distribution, tissue accumulation, and downstream therapeutic responses [28–30]. Our data add a preceding extracellular layer by showing that sPLA2-sensitive activity can reduce the EV signal recovered in recipient macrophages. In the LPS model, reduced macrophage-associated EV fluorescence indicates that inflammatory lung conditions can limit the accumulation of administered EVs in BALF macrophages. The partial restoration of this macrophage-associated EV signal by AZD2716 further supports the involvement of an sPLA2-sensitive process before or during cellular accumulation. Thus, extracellular fluid composition can influence recipient-cell accumulation before downstream cargo-dependent effects are evaluated.

Several limitations should be considered when interpreting these findings. First, macrophage-associated PKH-EV accumulation was used as the in vivo cellular readout associated with sPLA2-sensitive EV remodeling, but we did not directly determine whether larger or smaller EV fractions exert distinct protective or pro-inflammatory effects on lung inflammation, cytokine production, or chemokine expression. Second, because the analyzed material was a 10,000 × g EV-enriched fraction and size changes were primarily assessed by NTA-based particle-size profiling, these measurements do not distinguish intact EVs from co-isolated non-vesicular material, including debris, lipid fragments, or protein aggregates, and do not resolve subtype-specific EV remodeling. The direct lipid intermediates or membrane-level events underlying the size redistribution also remain unresolved. Third, because only male mice were used in this study, whether this sPLA2-sensitive EV remodeling axis operates similarly in female mice remains to be determined.

Future studies should define how broadly sPLA2-sensitive post-secretory EV stability operates across extracellular fluid environments and inflammatory conditions. Isoform-specific genetic approaches will be important for determining the contribution of endogenous sPLA2g5, because AZD2716 identifies an sPLA2-sensitive component but does not distinguish the contribution of individual sPLA2 isoforms. Studies combining sPLA2 inhibition with cargo-loaded EVs and orthogonal EV tracking approaches will further determine whether preserving extracellular EV stability improves productive cargo delivery and downstream biological responses.

## Conclusion

Our findings identify sPLA2 activity as an extracellular regulator of post-secretory EV stability. Across ex vivo and in vivo models, sPLA2-sensitive phospholipid hydrolysis was associated with EV size redistribution, AA generation, and reduced EV accumulation in recipient macrophages, with catalytic sPLA2g5 activity and AZD2716 sensitivity supporting this mechanism. These results position the extracellular fluid phase as an active regulatory layer in EV biology that can influence EV persistence and recipient-cell accumulation before downstream cargo-dependent effects emerge.

## Supplementary Information

Below is the link to the electronic supplementary material.

Supplementary Material 1

Supplementary Material 2

## Data Availability

All data generated or analysed during this study are included in this published article and its additional files.

## Abbreviations

AA: Arachidonic acid
AAV: Adeno-associated virus
ARDS: Acute respiratory distress syndrome
BALF: Bronchoalveolar lavage fluid
DPBS: Dulbecco’s phosphate-buffered saline
EV: Extracellular vesicle
UHPLC-MS: Ultra-high-performance liquid chromatography–mass spectrometry
LPS: Lipopolysaccharide
NTA: Nanoparticle tracking analysis
PBS: Phosphate-buffered saline
qPCR: quantitative real-time PCR
SEM: Scanning electron microscopy
sPLA2: secreted phospholipase A2
sPLA2g5: group V secreted phospholipase A2
WT: Wild-type
