# Supplementary material for: Secreted phospholipase activity alters the post-secretory stability of extracellular vesicles
Source: Biol Direct. 2026 Aug 17;21:239. doi: 10.1186/s13062-026-00937-7 (PMC13756863; doi:10.1186/s13062-026-00937-7)
Supplement: Supplementary file 1 [file 13062_2026_937_MOESM1_ESM.docx]

**Supplementary Fig. 1.** BALF cytospin morphology and differential cell counts after PBS or LPS exposure. Representative H&E-stained BALF cytospin images from PBS- and LPS-treated mice are shown with magnified views of the boxed regions. PBS-treated BALF contained predominantly macrophage-like cells with larger cell bodies and abundant cytoplasm, whereas LPS exposure induced prominent infiltration of smaller neutrophil-like cells with polymorphonuclear morphology. Blue arrows indicate macrophage-like cells, and red arrowheads indicate neutrophil-like cells. Macrophage-like and neutrophil-like cells were quantified per field based on cytospin morphology. BALF macrophages were distinguished based on cytospin morphology and selectively analyzed for PKH-EV accumulation in Fig. 4F and Fig. 5C. Data are presented as mean ± SD, n = 3 mice per group. Statistical significance was determined by unpaired two-tailed Student’s t-test. ***P < 0.005. Scale bars, 100 μm in overview images and 25 μm in magnified images.

**Supplementary Fig. 2.** PKH-only control for intratracheal EV tracking. Representative fluorescence images of BALF cytospin preparations after intratracheal administration of PKH-only control or PKH-labeled EVs are shown. The PKH-only control was prepared without EVs and subjected to the same dye incubation, centrifugation, washing, resuspension, and intratracheal administration procedures used for PKH-labeled EVs. BALF cells from the PKH-only control showed little detectable PKH fluorescence, whereas BALF cells from mice receiving PKH-labeled EVs showed clear cell-associated PKH signals. Nuclei were counterstained with DAPI. Scale bars, 100 μm.

**Supplementary Table 1**. Primer sequences used for quantitative real-time PCR.

| **Target gene** | **Forward primer (5′ to 3′)** | **Reverse primer (5′ to 3′)** |
| --- | --- | --- |
| *Actb* | CATTGCTGACAGGATGCAGAAGG | TGCTGGAAGGTGGACAGTGAGG |
| *Pla2g1b* | TCTCCCTCTTCTCCAATGCC | GAGAAACAAGAACAGAGAATCG |
| *Pla2g2a* | AGACCGGTGCTGTGTTACTC | CCCCTTGGTGGGAGTACTTG |
| *Pla2g2d* | GCTCTGGGCTGGAACTATGA | CCTGGGTTGCAGTTATACCG |
| *Pla2g2e* | ACAGGGACAGAGCTTGCAGT | TTCATCCTGGGGGAGGTAG |
| *Pla2g2f* | CCTGTCCTTCGTGGGCTAC | TTCCTGGTAGCAGCAGTCG |
| *Pla2g3* | GGCTGAGGCCACCTCATATACTTC | TCCTTTGCCCTCAGCACAGTCAAG |
| *Pla2g5*  WT-specific | GATTGGTGCTGTCAGATGCAC | AGGAGTCGTGTTCGCAGATG |
| *Pla2g5*  WT/Mut shared | ATGGCTTCTACGGCTGCTAC | AGGAGTCGTGTTCGCAGATG |
| *Pla2g10* | GCTACTCTCGGGCTCAGGAC | TTGGCATTTGTTCTCTGCTG |
| *AAV ITR* | GGAACCCCTAGTGATGGAGTT | CGGCCTCAGTGAGCG |

*Actb* was used as the internal control. The Pla2g5 WT-specific primer set was used to detect the AAV-sPLA2g5-WT construct, whereas the Pla2g5 WT/Mut shared primer set was used to detect both the AAV-sPLA2g5-WT and AAV-sPLA2g5-H67A constructs. The AAV ITR primer set was used for AAV genome titer determination.

**Supplementary Table 2. Exact P values and statistical information**

| **Figure** | **Panel** | **Readout** | **Comparison** | **P value** | **Statistical test** | **Adjustment** | **n** | **Statistical unit** |
| --- | --- | --- | --- | --- | --- | --- | --- | --- |
| Fig. 1 | D | BALF-derived EV size fraction | 0–200 vs 200–400 nm | 0.0185 | Repeated-measures one-way ANOVA | Multiple-comparison testing | n = 4 | Mouse-derived BALF EV preparation |
| Fig. 1 | D | BALF-derived EV size fraction | 0–200 vs 400–1,000 nm | 0.0505 | Repeated-measures one-way ANOVA | Multiple-comparison testing | n = 4 | Mouse-derived BALF EV preparation |
| Fig. 1 | D | BALF-derived EV size fraction | 200–400 vs 400–1,000 nm | 0.0008 | Repeated-measures one-way ANOVA | Multiple-comparison testing | n = 4 | Mouse-derived BALF EV preparation |
| Fig. 1 | G | 0–200 nm fraction | PBS vs BALF | 0.0153 | Paired two-tailed Student’s t-test | None | n = 6 | Mouse-derived EV preparation |
| Fig. 1 | G | 200–400 nm fraction | PBS vs BALF | 0.00686 | Paired two-tailed Student’s t-test | None | n = 6 | Mouse-derived EV preparation |
| Fig. 2 | F | 0–200 nm fraction | PBS vs sPLA2-V | 0.00583 | Paired two-tailed Student’s t-test | None | n = 6 | Mouse-derived EV preparation |
| Fig. 2 | F | 200–400 nm fraction | PBS vs sPLA2-V | 0.0271 | Paired two-tailed Student’s t-test | None | n = 6 | Mouse-derived EV preparation |
| Fig. 2 | I | 0–200 nm fraction | BALF vs BALF + AZD2716 | 0.0348 | Paired two-tailed Student’s t-test | None | n = 6 | Mouse-derived EV preparation |
| Fig. 2 | I | 200–400 nm fraction | BALF vs BALF + AZD2716 | 0.0164 | Paired two-tailed Student’s t-test | None | n = 6 | Mouse-derived EV preparation |
| Fig. 4 | C | WT-specific primer | AAV-vector vs AAV-sPLA2g5-WT | <0.0001 | Ordinary one-way ANOVA | Tukey’s multiple comparisons | n = 8, 8, and 7 | Mouse |
| Fig. 4 | C | WT-specific primer | AAV-sPLA2g5-WT vs AAV-sPLA2g5-H67A | <0.0001 | Ordinary one-way ANOVA | Tukey’s multiple comparisons | n = 8, 8, and 7 | Mouse |
| Fig. 4 | C | WT-specific primer | AAV-vector vs AAV-sPLA2g5-H67A | >0.9999 | Ordinary one-way ANOVA | Tukey’s multiple comparisons | n = 8, 8, and 7 | Mouse |
| Fig. 4 | C | WT/Mut shared primer | AAV-vector vs AAV-sPLA2g5-WT | 0.0002 | Ordinary one-way ANOVA | Tukey’s multiple comparisons | n = 8, 8, and 7 | Mouse |
| Fig. 4 | C | WT/Mut shared primer | AAV-sPLA2g5-WT vs AAV-sPLA2g5-H67A | 0.352 | Ordinary one-way ANOVA | Tukey’s multiple comparisons | n = 8, 8, and 7 | Mouse |
| Fig. 4 | C | WT/Mut shared primer | AAV-vector vs AAV-sPLA2g5-H67A | <0.0001 | Ordinary one-way ANOVA | Tukey’s multiple comparisons | n = 8, 8, and 7 | Mouse |
| Fig. 4 | E | 0–200 nm fraction | AAV-vector vs AAV-sPLA2g5-WT | 0.0233 | Ordinary one-way ANOVA | Tukey’s multiple comparisons | n = 8, 8, and 7 | Mouse |
| Fig. 4 | E | 0–200 nm fraction | AAV-sPLA2g5-WT vs AAV-sPLA2g5-H67A | 0.0468 | Ordinary one-way ANOVA | Tukey’s multiple comparisons | n = 8, 8, and 7 | Mouse |
| Fig. 4 | E | 0–200 nm fraction | AAV-vector vs AAV-sPLA2g5-H67A | 0.970 | Ordinary one-way ANOVA | Tukey’s multiple comparisons | n = 8, 8, and 7 | Mouse |
| Fig. 4 | E | 200–400 nm fraction | AAV-vector vs AAV-sPLA2g5-WT | 0.0482 | Ordinary one-way ANOVA | Tukey’s multiple comparisons | n = 8, 8, and 7 | Mouse |
| Fig. 4 | E | 200–400 nm fraction | AAV-sPLA2g5-WT vs AAV-sPLA2g5-H67A | 0.0791 | Ordinary one-way ANOVA | Tukey’s multiple comparisons | n = 8, 8, and 7 | Mouse |
| Fig. 4 | E | 200–400 nm fraction | AAV-vector vs AAV-sPLA2g5-H67A | 0.986 | Ordinary one-way ANOVA | Tukey’s multiple comparisons | n = 8, 8, and 7 | Mouse |
| Fig. 4 | F | Number of macrophages per field | AAV-vector vs AAV-sPLA2g5-WT | 0.823 | Ordinary one-way ANOVA | Tukey’s multiple comparisons | n = 4 per group | Mouse |
| Fig. 4 | F | Number of macrophages per field | AAV-sPLA2g5-WT vs AAV-sPLA2g5-H67A | 0.566 | Ordinary one-way ANOVA | Tukey’s multiple comparisons | n = 4 per group | Mouse |
| Fig. 4 | F | Number of macrophages per field | AAV-vector vs AAV-sPLA2g5-H67A | 0.896 | Ordinary one-way ANOVA | Tukey’s multiple comparisons | n = 4 per group | Mouse |
| Fig. 4 | F | PKH-EV⁺ area in macrophages (%) | AAV-vector vs AAV-sPLA2g5-WT | 0.0089 | Ordinary one-way ANOVA | Tukey’s multiple comparisons | n = 4 per group | Mouse |
| Fig. 4 | F | PKH-EV⁺ area in macrophages (%) | AAV-sPLA2g5-WT vs AAV-sPLA2g5-H67A | 0.0065 | Ordinary one-way ANOVA | Tukey’s multiple comparisons | n = 4 per group | Mouse |
| Fig. 4 | F | PKH-EV⁺ area in macrophages (%) | AAV-vector vs AAV-sPLA2g5-H67A | 0.976 | Ordinary one-way ANOVA | Tukey’s multiple comparisons | n = 4 per group | Mouse |
| Fig. 5 | B | Pla2g2a | PBS vs LPS | 0.207 | Unpaired two-tailed Student’s t-test | None | n = 6 per group | Mouse |
| Fig. 5 | B | Pla2g2d | PBS vs LPS | 0.000550 | Unpaired two-tailed Student’s t-test | None | n = 6 per group | Mouse |
| Fig. 5 | B | Pla2g3 | PBS vs LPS | 0.000382 | Unpaired two-tailed Student’s t-test | None | n = 6 per group | Mouse |
| Fig. 5 | B | Pla2g5 | PBS vs LPS | 0.00403 | Unpaired two-tailed Student’s t-test | None | n = 6 per group | Mouse |
| Fig. 5 | B | Pla2g10 | PBS vs LPS | 0.170 | Unpaired two-tailed Student’s t-test | None | n = 6 per group | Mouse |
| Fig. 5 | C | Number of macrophages per field | PBS/Veh vs PBS/AZD2716 | 0.3985 | Ordinary one-way ANOVA | Tukey’s multiple comparisons | n = 6 per group | Mouse |
| Fig. 5 | C | Number of macrophages per field | PBS/Veh vs LPS/Veh | 0.7825 | Ordinary one-way ANOVA | Tukey’s multiple comparisons | n = 6 per group | Mouse |
| Fig. 5 | C | Number of macrophages per field | PBS/Veh vs LPS/AZD2716 | >0.9999 | Ordinary one-way ANOVA | Tukey’s multiple comparisons | n = 6 per group | Mouse |
| Fig. 5 | C | Number of macrophages per field | PBS/AZD2716 vs LPS/Veh | 0.1025 | Ordinary one-way ANOVA | Tukey’s multiple comparisons | n = 6 per group | Mouse |
| Fig. 5 | C | Number of macrophages per field | PBS/AZD2716 vs LPS/AZD2716 | 0.3651 | Ordinary one-way ANOVA | Tukey’s multiple comparisons | n = 6 per group | Mouse |
| Fig. 5 | C | Number of macrophages per field | LPS/Veh vs LPS/AZD2716 | 0.8324 | Ordinary one-way ANOVA | Tukey’s multiple comparisons | n = 6 per group | Mouse |
| Fig. 5 | C | PKH-EV⁺ area in macrophages (%) | PBS/Veh vs PBS/AZD2716 | 0.9899 | Ordinary one-way ANOVA | Tukey’s multiple comparisons | n = 6 per group | Mouse |
| Fig. 5 | C | PKH-EV⁺ area in macrophages (%) | PBS/Veh vs LPS/Veh | 0.0077 | Ordinary one-way ANOVA | Tukey’s multiple comparisons | n = 6 per group | Mouse |
| Fig. 5 | C | PKH-EV⁺ area in macrophages (%) | PBS/Veh vs LPS/AZD2716 | 0.9695 | Ordinary one-way ANOVA | Tukey’s multiple comparisons | n = 6 per group | Mouse |
| Fig. 5 | C | PKH-EV⁺ area in macrophages (%) | PBS/AZD2716 vs LPS/Veh | 0.0151 | Ordinary one-way ANOVA | Tukey’s multiple comparisons | n = 6 per group | Mouse |
| Fig. 5 | C | PKH-EV⁺ area in macrophages (%) | PBS/AZD2716 vs LPS/AZD2716 | 0.9989 | Ordinary one-way ANOVA | Tukey’s multiple comparisons | n = 6 per group | Mouse |
| Fig. 5 | C | PKH-EV⁺ area in macrophages (%) | LPS/Veh vs LPS/AZD2716 | 0.0205 | Ordinary one-way ANOVA | Tukey’s multiple comparisons | n = 6 per group | Mouse |
