# Supplementary material for: Secreted phospholipase activity alters the post-secretory stability of extracellular vesicles
Source: Biol Direct. 2026 Aug 17;21:239. doi: 10.1186/s13062-026-00937-7 (PMC13756863; doi:10.1186/s13062-026-00937-7)
Supplement: Supplementary file 2 [file 13062_2026_937_MOESM2_ESM.docx]

**Supplementary Fig. 3.** Uncropped Western blot images corresponding to Fig. 4B. Uncropped Western blot images are shown for β-actin, GFP, and FLAG in HEK293T cells transfected with AAV-vector, AAV-sPLA2g5-WT, or AAV-sPLA2g5-H67A constructs. Dashed boxes indicate the cropped regions presented in Fig. 4B.
